# Passive and active screen time relate differently to attention in preschool children

**DOI:** 10.3389/fpsyg.2026.1737937

**Published:** 2026-03-18

**Authors:** Sigrid Hauge Nustad, Liv Merve Abrahamsson

**Affiliations:** Department of Psychology, University of Oslo, Oslo, Norway

**Keywords:** active screen time, attention, content, passive screen time, preschool children, review, screen exposure

## Abstract

The widespread integration of screen technology into daily life has increased screen exposure among preschool children aged three to five. However, the differential associations of passive and active screen time with attention in this age group remain underexplored. The distinction between passive and active screen time refers to the degree of interaction between the user and the screen content. This review synthesizes and compares findings on passive and active screen time to clarify whether they relate to attention in distinct ways. A targeted literature search yielded 13 relevant publications, comprising 10 empirical studies and three reviews. Findings point toward a negative association between passive screen time and attention. Conversely, active screen time correlates with improved bottom-up and orienting attention but weaker executive and top-down attention. Screen content may mediate these associations. Educational, interactive content may support attention, whereas evidence is mixed about whether fast-paced, passive content impairs it. Our results emphasize the need to distinguish both the type and content of screen time in evaluating associations with preschoolers' attention. We discuss methodological inconsistencies, outline theoretical and practical implications, and call for further research on emerging screen formats relevant to preschoolers.

## 1 Introduction

Screen technology has become an integrated part of young children's everyday lives. The World Health Organization recommends limiting screen time to no more than 1 h per day for children aged two to five (World Health Organization, 2019). Still, only 35.6% of children globally meet this guideline (McArthur et al., 2022). For over four decades, researchers have examined associations between children's screen-based media use and attentional capacities, yielding mixed results (Gueron-Sela and Gordon-Hacker, 2020; Kostyrka-Allchorne et al., 2017). As attention constitutes a core cognitive function, clarifying its relationship with screen time in childhood is essential for advancing theory, education, and practice. Traditionally, media effects research in children aged 5 and younger has focused on screen time (Barr et al., 2024). Screen time can be defined as any time spent looking at a screen (Jourdren et al., 2023). We argue that associations between attention and screen time likely vary by the type of screen use, and that treating exposure duration as a unitary construct may obscure critical distinctions. Grounded in previous research differentiating between passive and active screen time (Sweetser et al., 2012; Hu et al., 2020; Bal et al., 2024), this review evaluates whether different types of screen use exhibit distinct associations with attention.

Early work on screen time and attention has primarily examined children's television (TV) exposure (e.g., Zuckerman et al., 1978; Lorch et al., 1979). This literature largely reported negative associations between TV viewing and children's overall development and attention (e.g., Christakis et al., 2004; Johnson et al., 2007; Miller et al., 2007; Radesky and Christakis, 2016; Reid Chassiakos et al., 2016), although some studies failed to detect an association (e.g., Foster and Watkins, 2010; Ferguson, 2011). Subsequent research, however, began to distinguish between types of television content, showing that age-appropriate educational TV viewing can be associated with positive cognitive outcomes (e.g., Mares and Pan, 2013). Consistent with this differentiation, evidence from the preschool children suggest that television exposure may yield both beneficial and detrimental effects (Anderson et al., 2017). Content type, in particular, has emerged as a key moderator of the association between screen time and attention (Zimmerman and Christakis, 2007; Anderson et al., 2017).

Beyond TV, evidence on interactive screen use (e.g., video games) and the attentional outcomes is mixed, with reports of both negative associations and benefits for specific attentional capacities (Swing et al., 2010; Rothbart and Posner, 2015). To account for these inconsistencies, researchers have proposed a non-linear relationship between screen time and attentional subdomains (Liebherr et al., 2022). Studies using cumulative screen exposure scores, which aggregate across multiple screen types, generally report higher screen time to be associated with increased inattention problems (e.g., Tamana et al., 2019) and poorer focused attention (Gueron-Sela and Gordon-Hacker, 2020). More recent work further emphasizes the importance of screen interactivity in shaping attentional outcomes (Kirkorian et al., 2022; Webb et al., 2024). Media multitasking, now common in children's media use, shows weak negative associations with cognitive control and attention problems (Baumgartner et al., 2024).

Psychological research on attention is characterized by variation in its theoretical conceptualization but is generally approached in one of four ways: as endogenous (top-down) vs. exogenous (bottom-up) processes (e.g., Theeuwes, 1993); as selective (focused), sustained, divided, or alternating attention (Lezak et al., 2004); through Posner and Rothbart's (2007) orienting, alerting, and executive control systems; or as observable attention difficulties, such as hyperactivity and impulsive behavior. These theoretical perspectives on attention provide a structured framework for examining whether different forms of screen use may be differentially associated with specific attentional processes.

To differentiate different forms of screen engagement, we adopt the established categorization between *passive* and *active* screen time. In line with these prior conceptualizations, here we define *active screen time* as engagement in screen-based activities that require deliberate and ongoing user involvement to access, shape, or to generate content (Hu et al., 2020; Sweetser et al., 2012). Active screen time thus involves some form of cognitive, physical and/or social interaction through interactive use of devices, which allow for generating feedback in response to user input (Hu et al., 2020). Examples of active screen time include interactive computer, smartphone or tablet-based games, problem solving in educational applications, completing homework on a tablet, physically interactive video games such as those on the Nintendo Wii, or interactive communication via video calls. Conversely, *passive screen time* refers to time spent on screen-based activities that require little to no user interaction from the viewer once content delivery has commenced (Hu et al., 2020; Sweetser et al., 2012). Passive screen time is typically sedentary and involves mainly passive information intake, such as watching TV or movies (Sweetser et al., 2012).

Here, we focus primarily on preschool children aged 3–5 years. This choice is guided by several considerations: First, preschool age is categorized by rapid cognitive development and high plasticity (Jin and Lin, 2022; Rueda et al., 2004). Second, preschoolers' use of smart devices increases as mobile devices become more accessible and personalized (Lee et al., 2024). Given this group's increased screen use, clarifying the potential risks and benefits of screen use is crucial.

The present mini-review aims to address whether passive and active screen time are distinctly associated with attention in preschool children. Previous reviews have synthesized evidence across varying age ranges, media characteristics, and developmental outcomes. Many previous reviews examined attention as one of several developmental outcomes (e.g., Kirkorian et al., 2024; Anderson et al., 2017; Eirich et al., 2022; Bal et al., 2024) or examined specific subtypes of attention, such as selective attention (Dewi et al., 2025). Several reviews adopt a broader age range of interest (e.g., Kirkorian et al., 2024; Dewi et al., 2025; Anderson et al., 2017; Eirich et al., 2022; Santos et al., 2022). Many have specifically focused on the effects of television in preschoolers (e.g., Anderson et al., 1977; Thakkar et al., 2006; Namazi and Sadeghi, 2024) or across wider pediatric samples (e.g., Kostyrka-Allchorne et al., 2017). Some reviews have included multiple media sources (e.g., television, video games, cell phone) in preschoolers (Jourdren et al., 2023) or with broader age ranges (Santos et al., 2022), but often without differentiating or comparing different screen types. Although Bal et al. (2024) and Dewi et al. (2025) reference passive and active screen time, this distinction did not emerge as a primary analytic framework. In Bal et al. (2024), it appears as one of the several thematic dimensions within reviews of language development and executive functions. Similarly, Dewi et al. (2025) does not explicitly contrast passive and active screen engagement and focuses only on selective attention.

Overall, previous reviews on screen use and attention have largely focused on total screen time, either with broader thematic focus, age ranges, content or contextual factors, and without directly comparing passive and active screen time in preschool children. This mini-review addresses this gap by providing a focused and timely synthesis of evidence on active and passive screen time in relation to attention in preschool-aged children. While, as a mini-review, the present work does not provide an exhaustive analysis of the literature, the selected format enables a targeted examination of the studies addressing our research question within a rapidly evolving technological landscape. Given substantial shifts in children's media environment over the past 5–6 years (e.g., increased mobile and tablet use, digital media, and COVID-19 pandemic-related changes in screen exposure), emphasizing recent studies allows the review to reflect these contemporary patterns of screen use. Moreover, empirical work specifically linking screen use, attention, and preschool children has grown markedly since 2019. Accordingly, we synthesize evidence published between 2019 and 2025 on the relationship between children's attention and active vs. passive screen time and situate these findings within a cognitive psychology framework. Additionally, we explore mediating factors within these associations to elucidate the underlying mechanisms. By doing so, we aim to move the debate beyond broad screen time claims toward understanding how specific screen uses relate to children's attention.

## 2 Methods

A set of predefined criteria (see Supplementary material) was applied to ensure the relevance of the reviewed articles. To be included, articles needed to measure attention and include children's screen time as a factor. Studies were coded as either “active” or “passive” screen time in accordance with the operational definitions outlined in the Introduction. When primary studies did not explicitly adopt this framework, coding was based on the degree to which the activity required children to physically interact with the screens. Ambiguous cases were re-evaluated against operational definitions using a decision rule, whereby classification was determined by the extent to which the activity required interaction between the child and the screen device used. For example, passively watching a movie on a computer was coded as “passive” screen time despite the interactive capabilities of the computer. In studies where detailed descriptions of screen activities were not provided but instead assessed screen activity through parental reports of time spent on specific devices, activities were classified based on the level of interactivity associated with each screen activity, as described in the studies. For instance, where touchscreen tablet use was described in terms of online games or other interactive use, time spent on such tablets was coded as “active” screen time. Studies were excluded if they only measured “total screen time” and therefore could not be placed within either category. Coding was conducted by a single reviewer (see Supplementary material for coding procedure). The search was limited to peer-reviewed English-language studies published between 2019 and 2025 to reflect the rapid shifts in technological device use and screen exposure over the past 5–6 years, including increased use of mobile phones and tablets, the expansion of digital media, and a change in screen habits due to the COVID-19 pandemic. To identify potentially relevant literature, the databases Google Scholar, PubMed, and PsychInfo were searched between February and October 2025. The literature search included keywords related to attention (e.g., distract^*^, focus^*^, exogenous, endogenous, orient^*^, selective^*^, attent^*^, inattent^*^), children (e.g., child^*^, early childhood, preschool^*^, kid^*^, kindergarten), and screen exposure (e.g., screen^*^, media, digital, computer, mobile, smartphone, iPad, tablet, TV) combined using Boolean and adjacency operators. This search strategy was supported by screening relevant review papers. The full search strategy can be found in the Supplementary material. The review was restricted to children aged 3–5 years, and excluded those involving clinical populations (e.g., ADHD). This resulted in 13 included articles, including 10 empirical studies and three review articles.

The evidence was synthesized by categorizing the studies by two criteria: (1) type of screen use (passive vs. active screen use), and (2) direction of their association with attention (positive, negative, or null).

## 3 Results

An overview of how attention and screen time were defined and measured across the included studies is detailed in Table 1. Table 2 provides an overview of the outcomes reported in the empirical studies.

**Table 1:** Attention frameworks and measurements of attention and screen time across studies.

**References**	**Attention understood as**	**Measure of attention**	**Measure of screen time**
Corkin et al. (2021)	Inattention and hyperactivity	SDQ Inattention Hyperactivity subscale (mother report)	Parent-reported DCW
Hutton et al. (2019)	Top-down and bottom-up (dorsal/ventral networks)	fMRI functional connectivity	Story exposure (animated. Illustrated, audio) during fMRI
Jin and Lin (2022)	Alerting, orienting, executive attention	ANT-C: reaction times and accuracy	Parent-reported weekly tablet use
Jourdren et al. (2023)	Alerting, orienting, executive attention	Systematic review (varied tools)	Systematic review (varied tools)
Namazi and Sadeghi (2024)	Bottom-up and top-down	Systematic review (varied tools)	Exposure to fast/slow-paced and fantastical/realistic TV
Portugal et al. (2021)	Top-down vs. bottom-up, endogenous vs. exogenous	Eye tracking (gap-overlap and anti-saccade)	Parent-reported touchscreen use from toddlerhood to preschool age
Rose et al. (2022)	Inattention and selective attention	Observation during TV viewing, post-viewing problem-solving and attention tasks and CBQ	TV viewing (fast- vs. slow-paced) at home
Samson et al. (2021)	Sustained, selective, executive attention	ECAB	Parent-reported videogame hours per weekday
Santos et al. (2022)	Selective, sustained, alternated, divided	Systematic review (varied tools)	Systematic review (varied tools)
Twait et al. (2019)	Bottom-up and top-down attention	Orienting attention (TEA-Ch, Sky Search); Executive control (Symbol Search, WIPPSI)	6-week intervention: screen-recorded stories vs. dialogical reading
Vigil (2019)	Bottom-up and top-down processes	Parental reports using PKBS-2	Parent-reported portable screen time to the nearest half-hour
Zivan et al. (2019)	EEG-patterns of inattention	Resting-state EEG, Sky Search, and parent-reports (Conners and BRIEF)	6-week intervention: story-based video (screen) vs. live storytelling
Liu et al. (2021)	Alerting, orienting, executive attention networks	Eye tracking (gaze duration, fixation time/latency)	Experimental group: tablet-based educational games for 15 min/day, 12 weeks

This table outlines how the included studies conceptualized attention, the instruments or tasks used to measure it, and the corresponding measures of screen time.

DCW, Data Collection Worksheet; SDQ, Strengths and Difficulties Questionnaire; ANT-C, Attentional Network Task—Child Version; WIPPSI, Wechsler Preschool and Primary Scale of Intelligence; TEA-Ch, Test of Everyday Attention for Children; CBQ: Children's Behavior Questionnaire; ECAB, Early Childhood Attention Battery; PKBS-2, Preschool and Kindergarten Behavior Scale—Second Edition; BRIEF, Behavior Rating Inventory of Executive Function; fMRI, Functional Magnetic Resonance Imaging; EEG, Electroencephalography.

**Table 2:** Summary of empirical findings.

**Outcome**	**Finding**	**References**	**Passive/active screen use**
Positive correlation between screen time and attention	2 out of 10 articles showed positive correlation between screen time and attention	Liu et al., 2021 Samson et al., 2021	Active (educational tablet app) Active (recreational video games)
Negative correlation between screen time and attention	4 out of 10 articles showed negative correlation between screen time and attention	Zivan et al., 2019 Hutton et al., 2019 Twait et al., 2019 Vigil, 2019	Passive (screen-based story exposure) Passive (screen-based story exposure) Passive (screen-based story exposure) Active screen time (portable screen devices)
Both negative and positive correlations between screen time and attention	2 out of 10 articles showed both negative and positive correlation between screen time and attention	Jin and Lin, 2022 Portugal et al., 2021	Active (touchscreens) Active (touchscreens)
No correlation between screen time and attention	2 out of 10 articles showed no correlation between screen time and attention	Rose et al., 2022 Corkin et al., 2021	Passive (TV, fast vs. slow pace) Passive (TV) and Active (video games and portable devices)

The evidence from the included studies are organized according to the direction of the association of screen time with attention (positive, negative, or null), the number of studies reporting each type of association, corresponding sources, as well as the type of screen use (passive vs. active).

### 3.1 Passive screen time and attention

Consistent with the widely reported negative association between television viewing and attention in early childhood, forms of screen use that are passive have similarly been linked to less optimal attention outcomes. This might reflect screen time displacing activities important for attentional development, such as parent-child interaction, joint play, and outdoor play (Almeida et al., 2023). Neuroimaging evidence supports these behavioral findings. Zivan et al. (2019) used EEG to compare screen-based and in-person interactive storytelling and found higher functional connectivity in patterns associated with attention difficulties in the screen-based group (*F* = 17.27, *p* < 0.001, η^2^ = 0.40). These differences in EEG patterns significantly correlated with changes in parent-reported attention behavior (Conners: *r* = 0.39, *p* = 0.033; BRIEF: *r* = 0.41, *p* = 0.026). Similarly, Twait et al. (2019) compared screen-based storytelling and dialogic reading intervention in preschoolers using attention tests and EEG measures. The screen-based group showed poorer attention performance (*F* = 3.10 and 3.88, *p* < 0.05) and less efficient brain networks for orienting and executive control on EEG (*F* = 1.43, *p* = 0.02; *F* = 3.64, *p* = 0.05) than the dialogic reading group. Complementary fMRI evidence from Hutton et al. (2019), comparing preschoolers exposed to audio, illustrated, and animated stories, suggests that animated stimuli may tax working memory and reduce network integration supporting attentional reorienting and visual-language processing [DAN-L: −49%, VAN-VI (−47%), VAN-VP (−105%), *p* < 0.05]. However, some studies report no significant relationship between parental reports of passive screen time and inattention behavior in preschool children (Corkin et al., 2021).

Taken together, the neural patterns observed by Hutton et al. (2019), Zivan et al. (2019), and Twait et al. (2019) converge with behavioral findings from Almeida et al. (2023), suggesting that passive screen use may disrupt attentional network integration and foster observable inattention. Moreover, animated stimuli may bias attention toward visual perception, likely motivating children to continue seeking animated screen content (Hutton et al., 2019). This suggests that early exposure to screens may shape children's later screen-seeking tendencies, with potential implications for long-term attentional and behavioral outcomes.

### 3.2 Active screen time and attention

Unlike passive screen time, active screen time involves an element of interactivity that may engage attention differently. Evidence indicates mixed effects, associating active screen time with enhanced bottom-up processes, but weaker top-down control (Jin and Lin, 2022; Portugal et al., 2021; Samson et al., 2021). Greater exposure to active screen time may bias children's attention capacities toward rapid, bottom-up orienting at the expense of more effortful forms of attention control (e.g., Portugal et al., 2021).

Investigating the association between active screen time and observable attentional difficulties, Vigil (2019) found that portable screen use was negatively correlated with attention in older preschool children (*F* = 3.34, *p* < 0.05), accounting for 22.8% of the variance in attentional difficulties. In contrast, Samson et al. (2021) found that greater time spent playing recreational video games was associated with better performance on selective attention tasks (*rs* = 0.20, *p* < 0.05). Jin and Lin (2022) reported that greater tablet use was associated with higher accuracy (*r* = 0.28, *p* = 0.014) and faster reaction times (*r* = −0.27, *p* = 0.021) on tasks assessing altering and orienting attention, but poorer performance on measures of executive attention (*r* = 0.25, *p* = 0.029). In a similar study, Portugal et al. (2021) found that children with high touchscreen use showed greater attentional orienting but were slower to disengage compared to less-using children (Wald χ^2^ = 4.99, *p* = 0.026). Additionally, long-term use was associated with slightly poorer endogenous attention control (Wald χ^2^ = 3.95, *p* = 0.047). Interestingly, children who spent more time on touchscreens oriented faster to distractors before anticipating the target. This may be a corrective attentional strategy, with their faster orientation compensating for their poorer goal-directed and controlled attention (Portugal et al., 2021).

In contrast, one survey-based study reported null findings between active screen time and parent-reported inattention and hyperactivity behavior (Corkin et al., 2021). This finding may reflect methodological factors, including reliance on parent-reports and the operationalization of attention as inattention or ADHD-related behavior, which may fail to capture subtler attentional variations.

These findings indicate that active and passive screen time are likely differentially associated with distinct attentional capacities. Higher performance in selective and orienting attention has been observed in relation to interactive screen use, suggesting a tendency toward rapid responsiveness to stimuli and greater exogenous attention (Jin and Lin, 2022; Portugal et al., 2021; Samson et al., 2021). However, the decline in executive attention reported by Jin and Lin (2022) and the increases in inattention and overactivity observed by Vigil (2019) raise the question of whether these effects occur at the expense of goal-directed, focused attention. This attentional shift may shape how children deploy attention across contexts and reinforce rapid, stimulus-driven over deliberate and goal-directed attention. If so, active screen time during preschool years may have important implications for attentional development.

### 3.3 Mediating factors

This section explores mediating factors that may clarify the relationship between screen time and attention in preschool children.

#### 3.3.1 Passive screen time and pace of content

The pacing of visual stimuli (i.e., how quickly scenes or images change) during passive screen time appears to shape its relationship with attention, strengthening bottom-up attention while reducing prolonged concentration and top-down attention (Namazi and Sadeghi, 2024; Rose et al., 2022). Rose et al. (2022) revealed that faster-paced TV captured more visual attention (*F* = 35.54, *p* < 0.001, η^2^ = 0.48), whereas slower-paced TV supported sustained attention to a greater extent, particularly among younger children (*F* = 6.02, *p* = 0.019, η^2^ = 0.13). However, there was no significant difference in comprehension. This may indicate that children adapt their attention strategies to the program characteristics (Rose et al., 2022). A systematic review reported similar findings: in two of five relevant studies, watching faster-paced TV was associated with significantly poorer attention and more frequent shifts in attention during subsequent play, suggesting poorer top-down attentional control (Namazi and Sadeghi, 2024). However, findings are not unanimous, as some studies report no significant effect of pacing on attention (Namazi and Sadeghi, 2024; Rose et al., 2022).

#### 3.3.2 Active screen time and educational learning content

Engagement with educational content during active screen time may improve sustained attention, suggesting that some screen use may benefit preschool children's overall attentional capacities (Liu et al., 2021). Liu et al. (2021) found that 12 weeks of using an educational app improved children's performance on sustained attention tasks compared to the control group (η^2^ = 0.28, *p* < 0.0001), but there was small difference in orienting attention. In contrast, Vigil (2019) reported no significant effect of parental-reported educational screen use on the link between screen time and attention. These studies raise the possibility that educational apps may help foster children's sustained attention in ways that entertainment content does not. However, further research is needed to establish the generalizability of these effects.

## 4 Discussion

This review synthesized evidence on how distinct forms of screen time relate to attention in preschool children. Across studies, passive screen time is generally associated with poorer attentional outcomes, although the directionality of this relationship remains unclear. This result aligns with the broader literature indicating associations between TV viewing, a prototypical passive screen activity, and attention difficulties in early childhood (e.g., Christakis et al., 2004; Radesky and Christakis, 2016; Reid Chassiakos et al., 2016). In contrast, active screen time has been associated with improvements in bottom-up and orienting attention, and lower levels of top-down attention and attentional control. Some evidence suggests that educational content mitigates some of these negative effects and supports sustained attention in preschool children (Liu et al., 2021). The findings remain inconsistent regarding the role of fast-paced screen content, with some studies suggesting it may intensify the negative association, while others report negligible effects. Overall, it seems that attentional outcomes are associated not only with *how much* screen time children are exposed to, but also with *what kind of* content they engage with and *how* they interact with it. This is consistent with previous reviews highlighting media content and context as important dimensions for further investigation (Kirkorian et al., 2024; Dewi et al., 2025).

In our search, we observed inconsistencies across the studies, reflecting the variability in how both screen time and attention were defined and measured, particularly in the definition and assessment of attention. Across the 13 included papers, attention was defined using seven different frameworks and measured using eight distinct assessment methods, with parent-report measures encompassing six separate scales and questionnaires. Relatedly, in the literature, screen time was often treated as a single, undifferentiated measure representing total time spent on any digital device. Greater conceptual resolution (distinguishing between passive and active use, educational vs. recreational content, and device type) would enable more precise characterization of associations with developmental outcomes. Modern devices have the capacity to log activity-specific usage data. Several tools have been developed to support the integration of such data into research (Barr et al., 2020), alongside complementary approaches aimed at clarifying and refining screen time measures (e.g., Barr et al., 2024; Suh et al., 2024). These create new opportunities for increasingly precise measurements of screen habits.

Similarly, we noticed that the construct of attention was often inconsistently defined and measured. Table 1 provides a brief overview of how these constructs were understood and measured in the included studies. Some conceptualize attention in terms of attention problems, typically assessed through ADHD-related criteria such as observable inattention behavior and hyperactivity (Corkin et al., 2021). Earlier survey-based studies have similarly operationalized attention using such criteria and reported null associations (e.g., Foster and Watkins, 2010; Parkes et al., 2013; Peralta et al., 2018; Stevens and Mulsow, 2006), suggesting that measurement approach may contribute to the null or inconsistent findings. Others have also critiqued this approach, arguing that it captures overt difficulties and likely neglects subtler or domain-specific variations in attentional control (Jourdren et al., 2023). Overall, future research may benefit from greater conceptual and methodological precision by including clearer differentiation of screen activities, employing multi-dimensional attention assessments, and leveraging device-based usage data to enhance measurement validity and interpretability.

Furthermore, the literature frequently relies on parent-reported data for both screen time and attention. Parent-reports offer valuable insights but are also subject to potential bias. Given the controversies surrounding children's screen use, parents may over- or under-estimate their child's actual exposure. It should be noted that most studies employ cross-sectional or correlational designs, which preclude causal inferences. Jourdren et al. (2023) reports that although few studies examined directionality, most indicated a bidirectional relationship, with early attention difficulties predicting increased later screen use. Several mechanisms may explain this pattern. Children with pre-existing inattention may be more attracted to screen-based activities, while parental stress linked to challenging temperaments can further increase screen exposure (Almeida et al., 2023; Santos et al., 2022). Accordingly, current evidence cannot disentangle whether screen time affects attention or merely reflects pre-existing attentional vulnerabilities (i.e., greater screen use among children with weaker attentional capacities).

Although most studies reviewed here report statistically significant associations between screen time and attention, the magnitude of these associations varies considerably. Neuroimaging studies (e.g., Zivan et al., 2019; Hutton et al., 2019) found relatively strong effects for passive screen exposure (η^2^ = 0.21–0.40), whereas studies using parent-reports or behavioral checklists showed weaker (β = 0.14) or null associations (Corkin et al., 2021). In contrast, studies examining active screen use reported moderate (*r* = 0.28) to large (η^2^ = 0.28) positive effects on certain attention capacities (e.g., Jin and Lin, 2022; Liu et al., 2021). Overall, the strength and direction of effects differ by type of screen activity type and the measurement approach. Interactive and educational screen activities showed positive associations with moderate to large effects on specific attention capacities in preschool children.

It is important to note that no formal quality appraisal or risk-of-bias assessment was undertaken in this mini-review. Accordingly, the discussion of methodological strengths and limitations reflects interpretative comparison rather than systematic quality grading procedures typically employed in systematic reviews. Additionally, the reviewed literature predominantly characterizes screen interactivity in behavioral terms, with limited consideration of other dimensions such as cognitive engagement. It is also worth mentioning that the “active” screen use may include a broad range of qualitatively distinct forms of screen interaction, such as manipulative, embodied, or socially contingent engagement. The present review did not differentiate between these potential subcategories. These sub-categories may relate differently to attention.

### 4.1 Implications and future directions

This review suggests that preschool children's screen use requires a more complex conceptual framework than exposure duration alone. While research on passive screen use was generally associated with reduced attention, interactive and educational screen use tended to show more favorable attentional outcomes. Overall, the findings indicate that the passive-active distinction, as well as consideration of both the content and mode of engagement, may be theoretically useful. Moreover, the literature points to a potential bidirectional relationship between screen use and attention. Children showing early signs of inattention may be more drawn to screens, while high early exposure may relate to attentional differences. Thus, further research clarifying causal pathways and identifying vulnerable groups remain important.

From a practical perspective, caregivers and educators may consider not only duration but also the quality and interactivity of screen activities in preschool children. WHO guidelines recommend no more than 1 h of screen time per day for children aged two to five. Based on the present findings, differentiating between types of screen use may aid interpretation of these recommendations. A more differentiated understanding of screen engagement may therefore inform future guidance and research.

As shown in Table 1, the reviewed studies show considerable variability in the definitions and measurement approaches used for both screen time and attention. More consistent operationalization across studies may facilitate interpretation and cross-study comparisons. Longitudinal, ethically grounded experimental approaches (e.g., interventions that reduce screen time or structure its context and content) are useful to test the direction of the relationship between screen time and attention development in early childhood. Future meta-analyses could provide a quantitative synthesis of the findings across studies. Given the heterogeneity in definitions, frameworks, and methods, careful subgrouping and moderator analyses are needed.

Furthermore, the newer trends in preschoolers' screen use (e.g., exposure to fast-paced and algorithmically-curated content, “scrolling,” using multiple screen devices simultaneously, and “sludge videos”) remain largely overlooked. Given the limited empirical evidence on preschool children's screen use in digital environments, characterization of the emerging exposure patterns would support future research on attentional associations.
