# Supplementary material for: Passive and active screen time relate differently to attention in preschool children
Source: Front Psychol. 2026 Mar 18;17:1737937. doi: 10.3389/fpsyg.2026.1737937 (PMC13038535; doi:10.3389/fpsyg.2026.1737937)
Supplement: Supplementary file 1 [file Table_1.docx]

**Supplementary Material**

**Supplementary Table 1.** Search Strategy

| **Literature Search Strategy** | |
| --- | --- |
| **Category** | **Keywords** |
| Attention | *attention* OR *inattention* OR *(exogenous adj2 attent**) OR *(endogenous adj2 attent**) OR *(focus* adj2 attent*) OR *(sustain* adj2 attent*) OR *(select* adj2 attent*) OR *(divid* adj2 attent*) OR *(alternat* adj2 attent*) OR *(orient* adj2 attent*) OR *(alert* adj2 attent*) OR *(executive adj2 attent**) |
| Screen Time | *(screen* OR *media* OR *digital)* adj2 (*expos** OR *time* OR *use** OR *view** OR *skill**) OR *television* OR *TV* OR *mobile* OR *computer* OR *tablet* OR *iPad* OR *phone* OR *smartphone* OR *touchscreen* OR *app** OR *digital device* OR *gaming* |
| **Preschool Children** | *Child** OR *early childhood* OR *preschool* OR *preschoolers* OR *kid** OR *kindergarten* OR (*child** OR preschool*) adj (*development* OR *education*) OR *age** adj (3 OR three OR 4 OR four OR 5 OR five) |
| **Full Search Combination** | (*keywords for attention*) AND (*keywords for screen time*) AND (*keywords for preschool children*) |
| **Databases searched** | PsychINFO, Pubmed, Google Scholar |
| **Part of journals searched** | Search for terms in title, abstract, and keywords |
| **Years of search** | *2019-2025* |
| **Language** | English language only |
| **Types of studies to be included** | Peer-reviewed; empirical studies and reviews |
| **Inclusion criteria** | Human participants aged 3–5; Studies measuring both screen time and attention |
| **Exclusion criteria** | Clinical populations (e.g., ADHD);  studies reporting only “total screen time” without breakdown by type of screen use |
| **Date of last search** | October 3rd 2025 |

**Supplementary Table 2.** Coding Procedure

| **Criterion** | **Decision** | **Example** |
| --- | --- | --- |
| Did the study define screen use as “active” or “passive” consistent with our operational definitions? | Use authors’ classification | Study labels online gaming as active screen use |
| If not defined by the authors:   - Does the activity require direct interaction between the child and the screen (e.g., touching, responding)? | “Active” | Playing touchscreen games, interactive apps |
| If not defined by the authors:   - Does the activity not require interaction and involves primarily viewing or listening without contingent response | “Passive” | Watching TV or a streamed movie |
| Screen use without detailed activity description | Code according to the level of interactivity described in the study | Tablet use described as online gaming would be classified as “Active” |
| Device has interactive capabilities but activity itself is non-interactive | Code based on activity, not device | Watching a movie on a computer would be classified as “Passive” |
| Only “total screen time” reported without activity differentiation | Exclude from analysis | Total daily screen time |
